# Ethnic Differences in Survival after Breast Cancer in South East Asia

**DOI:** 10.1371/journal.pone.0030995

**Published:** 2012-02-21

**Authors:** Nirmala Bhoo-Pathy, Mikael Hartman, Cheng-Har Yip, Nakul Saxena, Nur Aishah Taib, Siew-Eng Lim, Philip Iau, Hans-Olov Adami, Awang M. Bulgiba, Soo-Chin Lee, Helena M. Verkooijen

**Affiliations:** 1 Julius Center for Health Sciences and Primary Care, University Medical Center, Utrecht, The Netherlands; 2 Dermatology Block, National Clinical Research Centre, Kuala Lumpur Hospital, Kuala Lumpur, Malaysia; 3 Saw Swee Hock School of Public Health, National University of Singapore, Singapore, Singapore; 4 Department of Surgery, Yong Loo Lin School of Medicine, National University of Singapore, Singapore, Singapore; 5 Department of Surgery, Faculty of Medicine, University of Malaya, Kuala Lumpur, Malaysia; 6 Department of Hematology Oncology, National University Cancer Institute, National University Health System, Singapore, Singapore; 7 Department of Epidemiology, Harvard School of Public Health, Boston, Massachusetts, United States of America; 8 Department of Medical Epidemiology and Biostatistics, Karolinska Institutet, Stockholm, Sweden; 9 Department of Social and Preventive Medicine, Faculty of Medicine, Julius Centre University of Malaya, University of Malaya, Kuala Lumpur, Malaysia; 10 Imaging Division, University Medical Center Utrecht, Utrecht, The Netherlands; Health Canada, Canada

## Abstract

**Background:**

The burden of breast cancer in Asia is escalating. We evaluated the impact of ethnicity on survival after breast cancer in the multi-ethnic region of South East Asia.

**Methodology/Principal Findings:**

Using the Singapore-Malaysia hospital-based breast cancer registry, we analyzed the association between ethnicity and mortality following breast cancer in 5,264 patients diagnosed between 1990 and 2007 (Chinese: 71.6%, Malay: 18.4%, Indian: 10.0%). We compared survival rates between ethnic groups and calculated adjusted hazard ratios (HR) to estimate the independent effect of ethnicity on survival. Malays (n = 968) presented at a significantly younger age, with larger tumors, and at later stages than the Chinese and Indians. Malays were also more likely to have axillary lymph node metastasis at similar tumor sizes and to have hormone receptor negative and poorly differentiated tumors. Five year overall survival was highest in the Chinese women (75.8%; 95%CI: 74.4%–77.3%) followed by Indians (68.0%; 95%CI: 63.8%–72.2%), and Malays (58.5%; 95%CI: 55.2%–61.7%). Compared to the Chinese, Malay ethnicity was associated with significantly higher risk of all-cause mortality (HR: 1.34; 95%CI: 1.19–1.51), independent of age, stage, tumor characteristics and treatment. Indian ethnicity was not significantly associated with risk of mortality after breast cancer compared to the Chinese (HR: 1.14; 95%CI: 0.98–1.34).

**Conclusion:**

In South East Asia, Malay ethnicity is independently associated with poorer survival after breast cancer. Research into underlying reasons, potentially including variations in tumor biology, psychosocial factors, treatment responsiveness and lifestyle after diagnosis, is warranted.

## Introduction

In contrast to the West, where breast cancer incidence rates have plateaued or even decreased [1], [2], the incidence of breast cancer is rapidly escalating in Asia. In China and India, breast cancer rates have increased by up to 30% over the last 10 years, while in Japan, Korea and Singapore, incidence rates have doubled or even tripled in the past few decades [3]. Studies in Western settings have implicated ethnicity as a predictor of survival following breast cancer [4]. However, the impact of ethnicity on survival after breast cancer in Asian settings has hardly been studied.

South East Asia, with distinct genetic, cultural and lifestyle diversity was recently highlighted as an emerging focus for global health [5]. In Malaysia and Singapore, multiethnic nations comprising 3 major ethnic groups namely Malays, Chinese and Indians [6], [7], age-standardized incidence rates of breast cancer differ substantially. The rate is highest among the Chinese followed by the Indians and the Malays [6], [7]. Whilst Malay women have the lowest incidence of breast cancer, there is also some evidence of poorer prognosis compared to their Chinese and Indian counterparts [8]. It remains unclear whether stage at diagnosis, tumor characteristics, or treatment explains the survival disparities.

Using a large multicenter hospital-based cohort of breast cancer patients from Malaysia and Singapore, we investigated the impact of ethnicity on survival after breast cancer, and possible mechanisms to explain the survival disparities.

## Methods

### Ethics statement

The NUS Breast Cancer Registry has received approval from the NUS Institutional Review Board (NUS-IRB). The University Malaya Breast Cancer Registry was approved by the University Malaya's Ethical Committee. This study was approved by the respective ethical committees in both institutions. As the study relies on non-identifiable registry-based data, the need to obtain informed consent was waived.

### Participants

We used data from the Singapore-Malaysia Breast Cancer Registry which currently encompasses 5,769 women. This multi-institutional breast cancer registry is a merger between the National University Hospital (NUH) Breast Cancer Registry, and the University Malaya Medical Center (UMMC) Breast Cancer Registry [9]. The NUH is a tertiary university hospital in the city state of Singapore. Its breast cancer registry includes all consecutive 2,449 women diagnosed with breast cancer between 1990 and 2007. Information has been collected retrospectively between 1990 and 1995 and prospectively from 1995 to 2007. UMMC is an academic tertiary hospital situated in the relatively affluent part of Kuala Lumpur (Malaysia) and caters to a predominantly middle class urban population. The UMMC Breast Cancer Registry is a prospective database of 3,320 consecutive women who were newly diagnosed with breast cancer between 1993 and 2007.

In the current study, we included all 5,264 women from the three major ethnic groups in Malaysia and Singapore i.e. Chinese, Malays and Indians, who were diagnosed with invasive breast cancer. Women of other ethnic minorities (n = 198) were excluded as were women with carcinoma in situ (n = 307).

### Study variables

The determinant of interest was ethnicity (Chinese, Malay, Indian), and the outcome was death from all causes. Data on patient characteristics included age at diagnosis, and center of treatment (NUH, UMMC). Patients were staged according to the 5^th^ edition of TNM American Joint Committee on Cancer (AJCC) system if diagnosed before January 1^st^ 2003, and according to the 6^th^ edition of AJCC if diagnosed after this date. Variables on disease characteristics included pathologically determined tumor size (available as continuous value and categorized into 0·1–20.0 mm, 20·1–50·00 mm, >50·0 mm, unknown), pathologically determined lymph node involvement (available as absolute number of positive nodes and categorized into yes, no, unknown), distant metastasis (detected by means of computed tomography of the thorax, abdomen and pelvis, and bone scan in patients with clinical stage III breast cancer; yes, no, unknown), estrogen receptor (ER) status/progesterone receptor (PR) status (positive when >10% of tumor cells stained positive during immunohistochemical testing, negative, unknown), and tumor grade (Scarff-Bloom-Richardson classification; grade 1, grade 2, grade 3, unknown). Loco-regional treatment was classified as no loco-regional treatment, complete loco-regional treatment (i.e. mastectomy, or breast conserving surgery [BCS] followed by radiotherapy), and incomplete treatment (i.e. BCS only, or radiotherapy only). Administration of chemotherapy, and hormone therapy were categorized as yes and no.

### Follow-up and outcome assessment

In both centers, patients were monitored via scheduled appointments in the specialist breast clinics. Data on mortality were obtained from the hospitals' medical records, as well as through active follow-up. In addition, vital status was verified through direct linkage with the National Registration Department in Malaysia. In this hospital based cancer registry, information on cause of death or cancer recurrence was not available for the majority of patients. Follow-up time was calculated as the interval between date of diagnosis and date of death, or date of last contact, whichever came first.

### Statistical analysis

All categorical variables were described by proportions and compared using the Chi square test. Continuous variables were expressed in medians and compared using the Kruskal Wallis test. Overall survival was estimated using Kaplan-Meier analyses and compared by log-rank test.

Cox regression analysis was performed to estimate the relative risk for all-cause mortality expressed as hazard ratio (HR) between women of different ethnicities. Time at entry was date of diagnosis with breast cancer, and exit time was date of death (from all causes), date at last contact, or Nov, 2010 (linkage with national mortality registry in Malaysia). This model was adjusted for age at diagnosis, center, year of diagnosis, tumor size, lymph node involvement, metastases, estrogen and progesterone receptor status, tumor grade, loco-regional therapy, chemotherapy, and hormone therapy.

Within the subgroup of patients with pathologically confirmed tumor size and lymph node status (N = 3,712), we studied possible effect modification by tumor size on the association between ethnicity and lymph node metastasis by including the interaction terms ‘ethnic groups multiplied by tumor size in mm’ into a logistic regression model with lymph node involvement as the outcome. Patients were subsequently grouped according to tumor size <20 mm, 20 to 50 mm, and >50 mm. Within each category, logistic regression analysis was used to determine the association between lymph node involvement (outcome) and ethnicity (predictor), adjusted for absolute tumor size (mm), tumor grade, ER status and PR status.

Two-tailed *p*-values below 0·05 and HRs with 95%CI which did not include 1·00 were considered as statistically significant. All analyses were performed using SAS version 9·1 (SAS Institute Inc, Cary, NC).

## Results

### Descriptive data

In this hospital based cohort of 5,264 multi-ethnic Asian women with breast cancer, there were 3,767 Chinese (71·6%), 968 Malays (18·4%) and 529 Indians (10·0%). Median age at diagnosis was 50 years (Table 1). Malay patients were significantly younger at diagnosis (median = 46 years) than Chinese (51 years) and Indian (53 years) women; *p*<0·001.

**Table 1:** Distribution of Patient Profile, Tumor Characteristics and Treatment According to Ethnicity in 5,264 Southeast Asian Women with Breast Cancer.

	Total	Chinese	Malay	Indian	*P* value^b^
	*N* = 5 264	*N* = 3 767	*N* = 968	*N* = 529	
	N (%)^a^	n (%)^a^	n (%)^a^	n (%)^a^	
**Median age at diagnosis**, years	50	51	46	53	<0·001
**Median tumor size**, mm^c^	30	25	35	30	<0·001
**Lymph node involvement**					<0·001
Absent	2 804 (54·7)	2 106 (57·1)	429 (46·4)	269 (52·0)	
Present	2 324 (45·3)	1 580 (42·9)	496 (53·6)	248 (48·0)	
Unknown	136	81	43	12	
**Estrogen receptor status**					0·004
Positive	2 436 (56·6)	1 825 (58·1)	407 (52·5)	231 (52·5)	
Negative	1 892 (43·4)	1 315 (41·9)	368 (47·5)	209 (47·5)	
Unknown	909	627	193	89	
**Progesterone receptor status**					0.172
Positive	1 960 (52·4)	1 469 (53·3)	321 (50·7)	170 (48·7)	
Negative	1 778 (47·6)	1 287 (46·7)	312 (49·3)	179 (51·3)	
Unknown	1 526	1 011	335	180	
**Tumor grade**					0.002
Good differentiation	466 (11·8)	375 (13·0)	57 (8·5)	34 (8·8)	
Moderate differentiation	1 864 (47·4)	1 368 (47·5)	313 (46·8)	183 (47·3)	
Poor differentiation	1 604 (40·8)	1 135 (39·4)	299 (44·7)	170 (43·9)	
Unknown	1 330	889	299	142	
**Loco-regional therapy** d					<0·001
Complete treatment	4 140 (90·2)	3 081 (92·7)	636 (79·8)	423 (90·2)	
Incomplete treatment	206 (4·5)	135 (4·1)	50 (6·3)	21 (4·5)	
None	243 (5·3)	107 (3·2)	111 (13·9)	25 (5·3)	
**Chemotherapy**					0·001
Yes	3 116 (59·2)	2 172 (57·7)	617 (63·7)	327 (61·8)	
No	2 148 (40·8)	1 595 (42·3)	351 (36·3)	202 (38·2)	
**Hormone therapy** e					<0·001
Yes	2 372 (85·6)	1 777 (86·5)	361 (79·0)	234 (89·7)	
No	400 (14·4)	277 (13·5)	96 (21·0)	27 (10·3)	

a Column percentage is presented except for center where row percentage is presented.

b Compared using χ^2^ test for categorical variables and Kruskal Wallis test for continuous variables.

c Absolute tumor size was only available in 4 359 patients i.e. in 80.4% of the Chinese, 89.2% of Malays and 88.5% of Indians.

d Only includes 4,589 patients with TNM stage I to stage III breast cancer. Complete treatment consists of mastectomy, or breast conserving surgery followed by radiotherapy. Incomplete treatment includes breast conserving surgery only or radiotherapy only.

e Only includes 2 772 patients with estrogen or progesterone receptor positive tumors.

The median tumor diameter was higher among Malay women (35 mm) than among Chinese (25 mm) and Indian (30 mm) women (*p*<0·001). Sixteen percent of Malay women were diagnosed with distant metastases (TNM stage IV) at presentation compared with 9% among Chinese and 4% among Indian women (Table 1). Chinese women were more likely than women of other ethnicities to present with stage I breast cancer and exhibit favorable tumor characteristics such as ER positivity (p = 0·002), PR positivity (p<0·001) or good differentiation (p<0·001).

Among women with non-metastatic breast cancer, the Malays were least likely to receive complete loco-regional treatment (Table 1). Within the subgroup of women who underwent breast surgery, a higher proportion of Malay women underwent breast conserving surgery compared to Chinese or Indian women (34·2% vs. 23·7% vs. 25·6%, respectively; p<0·001). Overall, Malay women were significantly more likely to receive chemotherapy compared to women from other ethnic groups (Table 1). Among women with estrogen receptor positive tumors, Malays were significantly less likely to receive hormonal therapy compared to the Chinese and Indians (79.1% vs. 87.3% vs. 90.9%; p<0.001).

### Survival

**Figure 1:**
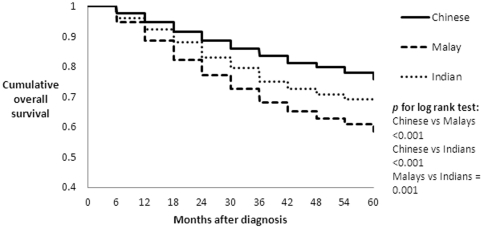
Cumulative overall survival by ethnicity in 5,264 South East Asian women with breast cancer.

After 30,882 person-years of follow-up, a total of 1,690 deaths from all causes had occurred. Five year overall survival was highest in the Chinese women (75·8%; 95%CI: 74·4%–77·3%) followed by Indians (68·0%; 95%CI: 63·8%–72·2%), and Malays (58·5%; 95%CI: 55·2%–61·7%) (Figure 1). Among patients with early breast cancer (stage I and II), the Chinese also had the highest overall survival (88·1%; 95%CI: 86·8%–88·3%), whereas survival was not significantly different between the Indians (80·5%; 95%CI: 76·1%–84·9%) and the Malays (77·5%; 95%CI: 73·8%–81·2%). In the more advanced stages (stage III & IV), the survival of the Chinese and Indian patients did not differ significantly (43·7%; 95%CI: 40·4%–47·0% and 43·3%; 95%CI: 35·5%–51·1%, respectively), whereas the Malays had significantly lower survival (34·4%; 95%CI: 29·6%–39·2%) than the Chinese.

Following adjustment for age at diagnosis, calendar year, and center, Malay patients with breast cancer were associated with substantially increased risk of death as compared to their Chinese counterparts whereas, Indian ethnicity was associated with moderately increased risk (Table 2). These mortality risks further attenuated when we adjusted for tumor size at presentation, which may be regarded as a proxy of health awareness in our population; HR for Malays versus Chinese: 1·66; 95%CI: 1·48–1·86, and HR for Indians versus Chinese: 1·14; 95%CI: 0·98–1·33. In a full model adjusted for socio-demographic factors, tumor characteristics and treatment, Malay ethnicity remained independently associated with risk of mortality following breast cancer compared to the Chinese (HR: 1.34; 95%CI: 1.19–1.51). However, there was no significant difference in mortality between the Chinese and Indian breast cancer patients.

**Table 2:** Association between Ethnicity and All-Cause Mortality Following Diagnosis with Breast Cancer in 5,264 Southeast Asian Women.

	Chinese *N* = 3 767	Malays *N* = 968	Indians *N* = 529
Hazard Ratio	1.00	1·90	1·24
(95%CI)^a^		(1·70–2·13)	(1·06–1·44)
Hazard Ratio	1.00	1·45	1·15
(95%CI)^b^		(1·29–1·63)	(0·98–1·34)
Hazard Ratio	1.00	1.34	1.14
(95%CI)^c^		(1·19–1·51)	(0.98–1.34)

a Estimated from Cox regression model adjusted for age at diagnosis, center, and year of diagnosis.

b Estimated from Cox regression model adjusted for age at diagnosis, center, year of diagnosis, tumor size, lymph node involvement, distant metastasis, estrogen receptor status, progesterone receptor status, and tumor grade.

c Estimated from Cox regression model adjusted for age at diagnosis, center, year of diagnosis, tumor size, lymph node involvement, distant metastasis, estrogen receptor status, progesterone receptor status, tumor grade, loco-regional therapy, chemotherapy, and hormone therapy.

### Tumor size and axillary metastases

It was found that tumor size significantly modifies the association between Malay ethnicity and axillary lymph node involvement (p for interaction = 0.028) but not for Indian ethnicity (p = 0.603). Within the subgroup of tumors below 20 mm, Malay patients were significantly more likely to have lymph node involvement compared to the Chinese and Indian women with breast cancer (35·4% versus 23·0% versus 17·4%, respectively; p = 0·004) (Table 3). Malay ethnicity remains significantly associated with an increased risk of axillary lymph node metastasis compared to the Chinese after adjustment for other tumor characteristics (adjusted OR 1·57, 95% CI: 1·05–2·37). In tumors measuring 20–50 mm, Malay patients were also more likely to have lymph node involvement (adjusted OR: 1·45; 95%CI: 1·15–1·84).

**Table 3:** Association between ethnic groups and lymph node involvement by tumor size in 3,712 Asian women with breast cancer.^a^

	Ethnicity			*P* value^b^
	Chinese	Malay	Indian	
**Tumor size less than 20 mm (N = 937)**				0.004
No nodal involvement, N (%)	568 (77·0)	84 (64·6)	57 (82·6)	
Lymph node involvement, N (%)	170 (23·0)	46 (35·4)	12 (17·4)	
Adjusted odds ratio for lymph node involvement^c^ (95% confidence interval)	1.00	1·57 (1·05–2·37)	0·64 (0·33–1·24)	
**Tumor size 20–50 mm (N = 2,228)**				0.002
No nodal involvement, N (%)	865 (53·8)	160 (43·7)	125 (49·0)	
Lymph node involvement, N (%)	742 (46·2)	206 (56·3)	130 (51·0)	
Adjusted odds ratio for lymph node involvement^c^ (95% confidence interval)	1.00	1·45 (1·15–1·84)	1·18 (0·89–1·55)	
**Tumor size more than 50 mm (N = 547)**				0.215
No nodal involvement, N (%)	105 (30·9)	34 (25·2)	16 (2·2)	
Lymph node involvement, N (%)	235 (69·1)	101 (74·8)	56 (77·8)	
Adjusted odds ratio for lymph node involvement^c^ (95% confidence interval)	1.00	1·40 (0·88–2·23)	1·69 (0·92–3·13)	

a Only including patients with pathologically confirmed tumor size and lymph node status.

b Using Chi Square test.

c Logistic regression model adjusted for absolute tumor size (mm), tumor grade, estrogen receptor status, and progesterone receptor status.

## Discussion

With this study, we have shown marked ethnic differences in disease presentation, treatment patterns, and survival of breast cancer patients in South East Asia. Women of Malay ethnicity presented at more advanced stages of breast cancer than women belonging to other ethnic groups. The Malays also seem to have a more aggressive tumor biology compared to the Chinese breast cancer patients. Furthermore, Malay ethnicity is associated with higher risk of death following breast cancer, even after accounting for demographic factors, tumor characteristics and treatment.

To our knowledge, this is the first large study to shed light on the impact of ethnicity on the survival of women following breast cancer in the Asian context. Strengths of our study include detailed information on tumor characteristics and treatment profile which are important prognostic determinants of survival related to breast cancer. Nevertheless, information on HER2 status was lacking, and we are unsure of its influence on the study findings. We also did not have adequate information on the causes of death of patients making it impossible to study breast cancer specific survival. We acknowledge that ethnic differences in co-morbidity and life expectancy may partly explain the observed ethnic disparities in survival. Chinese women in South East Asia have the highest life expectancy at birth (e.g. 77·1 years) whereas the Malay (73·6 years) and Indian (72·1 years) women have a slightly lower life expectancy [10], [11]. Nevertheless, these differences in life expectancy are unlikely to completely explain our results. The relationship between ethnicity and breast cancer survival seems complex and a variety of factors have been proposed to explain the ethnic disparities in breast cancer survival [4].

### Ethnic differences in socio-economic status and cultural values

Ethnicity has often been considered a proxy for socio-economic status [12], whereby a low socio-economic status has been linked to late stage at diagnosis with cancer [13], unequal access to optimal treatment [14], and poorer treatment adherence [15]. In our population, the Chinese have the highest household income and are most likely to receive tertiary education, whereas the Malays have the lowest income and education status, and the Indians fall in between [16], [17]. In the current study, Malay women presented at later stages of cancer and were less likely to receive complete loco-regional therapy and hormone therapy. Although socioeconomic status is a strong determinant of outcome in many chronic diseases, it is nevertheless unable to fully explain ethnic disparities in survival [18], [19].

In addition to socio-economic differences, we also see religious differences in our three ethnic groups, whereby the Malays are mostly Muslims, the Chinese are either Buddhists or Christians, and the Indians are mainly Hindus [16]. As religion and culture is intertwined, it is likely that psychosocial and cultural factors such as folk/religious beliefs, relationships with men, perceived risk, and beliefs in various treatments for breast cancer, may have an impact on disease awareness, access to early detection and thus stage at presentation of breast cancer [19]. Corroborating these, the emerging themes from a study in Malaysian women who presented at late stage breast cancer were cancer fatalism (i.e. the belief that death is inevitable when cancer is present), and use of alternative therapy [20]. Besides influencing stage at diagnosis, the above factors may also influence treatment acceptance and adherence [21].

### Ethnic differences in tumor biology

Ethnic variations in tumor biology have been reported, whereby certain ethnic groups are more likely to have unfavorable cancer types such as hormone receptor negative tumors, HER2 over-expression, basal-like breast tumors, or high grade tumors [22], [23]. In our study, Malay patients had more aggressive tumors compared to other races, as indicated by their higher risk of axillary lymph node metastasis at similar tumor sizes. Moreover, Malay and Indian women were more likely to have unfavorable tumor characteristics such as hormonal negative and high grade tumors. Adjustment for tumor related factors substantially attenuated the excess mortality in Indian patients but did not eliminate it completely among the Malays. As full characterization of breast cancer by molecular subgroups was not carried out in this study, we are unsure of its impact on our findings.

### Ethnic differences in response to treatment

There may be ethnic differences in tolerability and response to hormonal treatment and cytotoxic chemotherapy in breast cancer [24]. For instance, the activities of the CYP P450 group of enzymes which are responsible in metabolizing anti-hormonal drugs seem to vary between ethnic groups, due to underlying differences in functional polymorphisms [24]. Hence, anti-hormonal drugs such as tamoxifen may be more effective in certain ethnic groups. Furthermore, genotype-phenotype associations have been suggested based on studies among Chinese, Malay and Indian patients receiving doxorubicin [25], [26]. Compared to other ethnic groups Chinese may be predisposed to higher concentrations of doxorubicin associated with higher frequencies of polymorphism within the SLC22A16 gene [25], or CBR3 gene [26]. Certain anticancer therapies may therefore be more effective in the Chinese.

### Ethnic differences in lifestyle

Lifestyle factors, such as diet and body weight, are increasingly being recognized as important prognostic factors of breast cancer [4]. Owing to differences in religious and cultural practices, lifestyle profiles do differ substantially between the ethnic groups in South East Asia. In terms of diet, Malay women, for instance, are less likely to consume alcohol, whereas the Chinese women have a high intake of soy and consume the lowest amount of dietary fat [27]. A study conducted in China suggested that dietary practices such as increased soy intake are associated with decreased risk of death and recurrence among breast cancer survivors [28].

We also see important differences in prevalence of overweight and obesity in our three ethnic groups. Obesity is more common in Malay and Indian women, whereas the Chinese have the lowest body mass index [29], [30]. Obesity has been linked to late stage at presentation of breast cancer as well as substandard diagnostic work-up [31]. In addition, body weight and weight gain after the diagnosis of breast cancer have also been implicated in prognosis of breast cancer [32], which might explain some of the excess mortality among Malay and Indian women.

Our findings highlight the problem of late presentation among the Malays. As psychosocial and cultural factors, as well as socioeconomic status may contribute to delayed presentation [19], [20], culturally-sensitive programs and oncology practices are needed to improve breast health literacy in South East Asia. Such programs should aim to encourage early detection of breast cancer especially among the Malay women via participation in cancer screening activities such as self-breast examination, clinical breast examination, and mammographic screening. Further research is needed in Asian women to investigate tumor biology, ethnic differences in genetic variants associated with response to anticancer therapy and impact of cultural and lifestyle determinants on breast cancer survival.

In conclusion, Malay ethnicity is significantly associated with poorer survival following the diagnosis of breast cancer. The underlying reasons for this association are unclear but maybe explained by variations in tumor biology, psychosocial and cultural beliefs, susceptibility to anticancer treatment and lifestyle after diagnosis of breast cancer.
